# Analysis of Population Structure: A Unifying Framework and Novel Methods Based on Sparse Factor Analysis

**DOI:** 10.1371/journal.pgen.1001117

**Published:** 2010-09-16

**Authors:** Barbara E. Engelhardt, Matthew Stephens

**Affiliations:** 1 Computer Science Department, University of Chicago, Chicago, Illinois, United States of America; 2 Department of Statistics and Department of Human Genetics, University of Chicago, Chicago, Illinois, United States of America; University of Arizona, United States of America

## Abstract

We consider the statistical analysis of population structure using genetic data. We show how the two most widely used approaches to modeling population structure, admixture-based models and principal components analysis (PCA), can be viewed within a single unifying framework of matrix factorization. Specifically, they can both be interpreted as approximating an observed genotype matrix by a product of two lower-rank matrices, but with different constraints or prior distributions on these lower-rank matrices. This opens the door to a large range of possible approaches to analyzing population structure, by considering other constraints or priors. In this paper, we introduce one such novel approach, based on sparse factor analysis (SFA). We investigate the effects of the different types of constraint in several real and simulated data sets. We find that SFA produces similar results to admixture-based models when the samples are descended from a few well-differentiated ancestral populations and can recapitulate the results of PCA when the population structure is more “continuous,” as in isolation-by-distance models.

## Author Summary

Two different approaches have become widely used in the analysis of population structure: admixture-based models and principal components analysis (PCA). In admixture-based models each individual is assumed to have inherited some proportion of its ancestry from one of several distinct populations. PCA projects the individuals into a low-dimensional subspace. On the face of it, these methods seem to have little in common. Here we show how in fact both of these methods can be viewed within a single unifying framework. This viewpoint should help practitioners to better interpret and contrast the results from these methods in real data applications. It also provides a springboard to the development of novel approaches to this problem. We introduce one such novel approach, based on sparse factor analysis, which has elements in common with both admixture-based models and PCA. As we illustrate here, in some settings sparse factor analysis may provide more interpretable results than either admixture-based models or PCA.

## Introduction

The problem of analyzing the structure of natural populations arises in many contexts, and has attracted considerable attention. For example, methods for analyzing population structure have been used in studies of human history [1], [2], conservation genetics [3], domestication events [4], and to correct for cryptic population stratification in genetic association studies [5]–[7].

Two types of methods for analyzing population structure have become widely used: methods based on admixture models, such as those implemented in the software packages *structure* [6], [8], FRAPPE [9], SABER [10], and admixture [11]; and principal components analysis (e.g., [7], [12]), such as is implemented in the program SmartPCA [13]. In admixture-based models each individual is assumed to have inherited some proportion of its ancestry from each of 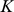 distinct populations. These proportions are known as the *admixture proportions* of each individual, and a key goal of these methods is to estimate these proportions and the allele frequencies of each population. Principal components analysis (PCA) can be thought of as projecting the individuals into a low-dimensional subspace in such a way that the locations of individuals in the projected space reflects the genetic similarities among them. For example, when the population structure conforms to a simple isolation-by-distance model with homogeneous migration then PCA effectively recapitulates the geographic locations of individuals [14], [15].

At first sight, these two different approaches to analysis of population structure appear to have little in common. For example, admixture-based methods involve an explicit model, whereas PCA, as usually described, does not. In this paper we describe how these approaches can be viewed within a single unifying framework. Specifically, they are both examples of low-rank matrix factorization with different constraints on the factorized matrices (e.g., [16]). Motivated by this general view we also consider a new method for analyzing population structure, sparse factor analysis (SFA), which lies in this same model class. We perform parameter estimation for SFA using a version of the expectation maximization (EM) algorithm, enabling application of SFA to genome-wide data.

We compare and contrast these three different methods on a range of real data and simulated examples. We find that SFA produces similar results to admixture-based models when the data conform to discrete and admixed populations, and can produce results similar to PCA when allele frequencies vary continuously with geography. Placing these different methods into a single framework also greatly aids comparisons among the methods, and provides helpful insights into why they may produce different results in practical applications.

### Population structure via low-rank matrix factorization

**Figure 1:**
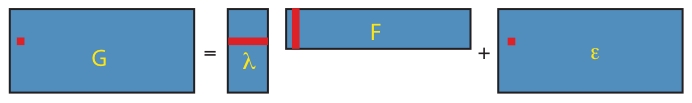
Low-dimensional matrix factorization via factor analysis. Each matrix in Equation 1 is illustrated by a blue rectangle and labeled. As in Equation 2, a single element of genotype matrix 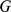, 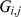 is shown in red, and is computed from the product of the appropriate factor loading and factor vectors plus the corresponding random error term (all highlighted in red).

In this section, we describe how admixture-based models and PCA can be viewed as factorizing an observed genotype matrix 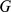 into a product of two low-rank matrices. We assume that 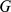 contains the genotypes of 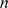 individuals at 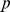 SNPs with genotypes coded as 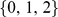 copies of a reference allele. Then both admixture-based models and PCA can be framed as models in which:
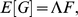
(1)or, equivalently,
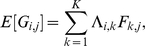
(2)where 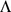 is a 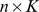 matrix and 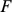 is a 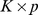 matrix, where 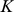 is typically small (Figure 1) (see Table 1 for a complete list of terms and constraints). In this framework, the primary difference between the approaches lies in the constraints or prior distributions placed on matrices 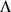 and 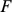 as follows.

**Table 1:** Relationship of terms in PCA, SFA, and admixture-based models.

		PCA	SFA	Admixture-based model
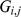	name	genotype matrix	genotype matrix	genotype matrix
	constraints	none	none	non-negative, integer valued
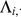	name	PCA loadings	factor loadings	admixture proportions for individual 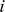
	constraints	orthogonal	none	non-negative, sum to one
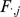	name	PCA factors	factors	twice mean allele frequencies for locus 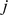
	constraints	orthonormal	variance is one	non-negative, in range 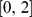
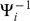	name	residual variance	residual variance	residual variance
	constraints	same for all 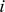 , 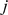	one for each 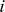	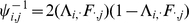

#### Admixture-based models

Under admixture-based models (as found in, e.g., *structure* [17] and related work), explicitly marginalizing the multinomial latent variables representing individual- and SNP-specific ancestry, 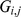 is assumed to be distributed as binomial 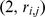, with 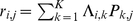, where 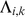 is the admixture proportion of individual 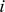 in population 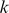 and 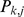 is the allele frequency of the reference allele in population 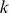. It follows that 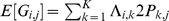, as in Equation 2 above with 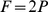. Thus, admixture-based models can be viewed as performing the matrix factorization (Equation 1) with the following constraints on 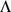 and 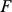: the elements of 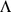 are constrained to be non-negative with each column summing to one; the elements of 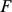 are constrained to lie within 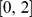. In Bayesian applications of this model, priors are placed on 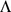 and 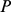, which can be thought of as imposing additional “soft” constraints on the matrices.

#### Principal component analysis

PCA can be derived by considering the model 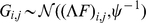. Specifically, consider maximizing the likelihood of this model with respect to parameters 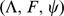, subject to the constraints: i) the 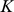 columns of 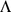 are orthogonal (so 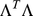 is diagonal); ii) the 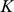 rows of 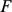 are orthonormal (so 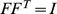). Then the columns of 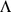 and rows of 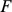 give the principal components (PCs) and the corresponding PC loadings. To see this, consider performing the constrained optimization via singular value decomposition (SVD) of 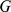: if 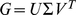 is the SVD for 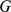, then setting 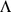 to the first 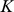 columns of 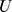 and 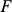 to the first 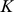 rows of 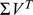 satisfies the constraints and maximizes the likelihood (by standard results on optimality of the SVD; e.g., [18]). However, PCA can be performed in exactly the same way, and so the result follows.

**Figure 2:**
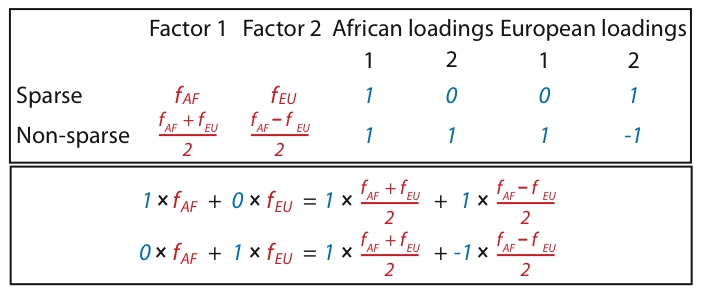
Illustration of two different ways that African and European individuals could be represented. In the first (sparse) representation in the first row, the factors (shown in red) each represent the mean allele frequencies for either the African population (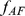) or the European population (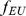); this lends to sparse loadings (shown in blue) for each individual, since the African individuals are only loaded on the factor representing the African population, and likewise for the European individuals. In the second (non-sparse) representation in the second row, each factor is a combination of 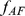 and 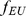, and each individual is loaded onto both factors. Note that the representations are equivalent by the equations under the table. Whereas SFA and admixture-based models tend to choose the first representation because of the sparse priors and implicit regularization, PCA tends towards the second representation (although the actual factors depend on other features of the data such as sample sizes of both groups).

Placing these two approaches to the analysis of population structure within a single framework helps illuminate some of their similarities and differences. For example, we can view both methods as attempting to approximate each individual's genotype vector by a linear combination of allele frequencies (Figure 2 illustrates different but equivalent linear combinations), but the admixture-based models are more restrictive because they insist on this linear combination being a *convex* combination (the admixture proportions must be non-negative and sum to one). This restriction makes sense if the study individuals conform closely to this assumption – that is, if each individual is indeed an admixture of a small number of ancestral populations – and in this case imposing this restriction leads to improved interpretability (each factor in 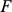 corresponds to the allele frequencies of an ancestral population). On the other hand, where the study individuals do not conform closely to this assumption, such as in isolation-by-distance models considered later, the less restrictive approach of PCA may enable the representation of a wider range of underlying structure.

Furthermore, viewing both methods within the framework of matrix factorization immediately suggests many alternative approaches to analyzing population structure. By modifying the constraints or priors on the matrices, one may hope to develop better methods for different latent structures. To illustrate this possibility, we consider here a version of sparse factor analysis (SFA) where the key idea is to encourage the 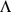 matrix to be sparse, attempting to represent each individual as a linear combination of a *small* number of underlying factors, without constraints (e.g., orthogonality) on the factors. Intuitively, sparsity can lead to more interpretable results than PCA, while the use of general linear combinations (and not only convex combinations) maintains flexibility in capturing a wider range of underlying structures. There are several different approaches to SFA (e.g., [19]–[22]); here we use a novel approach described below. Other possible methods for matrix factorization that may be appropriate for this problem include non-negative matrix factorization [23], and sparse PCA (e.g., [24]). We summarize results from these methods in our Discussion.

#### Sparse factor analysis

We now briefly describe our novel approach to SFA; see Methods for further details. The SFA model assumes 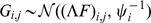, and encourages sparsity in the 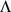 matrix by putting a prior on its elements (thus sparsity is a “soft” constraint, rather than a hard requirement). Specifically we use the *automatic relevance determination* (ARD) prior [25]–[27], which assumes 
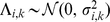
 where the variances 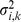 are hyper-parameters that are estimated by maximum likelihood. If the data are consistent with a small absolute value of 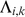 then 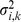 will be estimated to be small, which results in strong shrinkage of 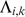 towards zero, inducing sparsity where it is consistent with the data. To ensure identifiability we constrain the rows of 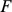 to have unit variance, which effectively determines the scale of the columns of 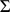; other than this we place no orthogonality constraints or prior distributions on 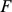 (unlike most applications of factor analysis; see also [28]).

## Results

We use simulated and real human genotype data to compare and contrast SFA, PCA, and an admixture-based model, admixture [11]. (admixture typically produces results that are qualitatively similar to the results from *structure*, but is computationally more convenient for large data sets.) In particular, we will compare the matrices 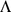 and 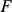 produced by each method (see above) in a variety of settings. For consistency of terminology we will refer to the columns of 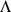 as the *loadings* and the rows of 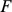 as the *factors* for each method. Because each method scales the absolute values of the factors (and loadings) in different ways, the absolute values of the factors (and loadings) are not comparable across methods, but the relative values are. Thus, when looking at the figures to follow, differences in the scales of the axes for different methods are irrelevant and should be ignored. A summary of the results with simple interpretations is in Table 2.

**Table 2:** Summary of results across PCA, SFA, and admixture-based models.

	PCA	SFA	SFAm	Admixture model
HapMap	mean + 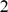 contrasts	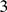 pop means	NR	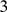 pop means
1-D habitat	mean + 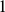 contrast	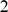 ends of line	mean + 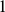 contrast	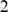 ends of line
2-D habitat	mean + 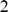 contrasts	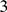 contrasts	mean + 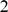 contrasts	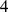 corners of square

The columns are the four different types of matrix factorizations we considered, and the rows are the different data sets we applied each method to that show easily interpretable results. “NR” indicates that we did not run the method on those data, and a ‘–’ indicates that the results were not straightforward to describe (see Results for details). *Mean* indicates that the factor is the mean allele frequencies for the complete set of individuals; *contrast* indicates a difference in the allele frequencies along a geographical gradient.

For PCA we follow the common practice (e.g., as in SmartPCA [13]) of first mean-centering the columns of 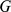 and standardizing them to have unit variance before applying PCA. This slightly complicates comparisons across methods because, formally, we are using PCA to factorize a different matrix than the other two methods. However, the results of PCA on the standardized matrix actually imply a factorization of the original matrix, but with one additional factor and corresponding loading. Specifically, the additional factor corresponds to the vector of genotype means and the additional loading corresponds to a vector of ones (see Text S1). To aid comparisons among the methods we explicitly include this additional factor and loading in the figures and discussions.

### Discrete and admixed populations

For simplicity we begin by applying the methods to a small data set of 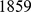 SNPs typed on 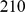 unrelated HapMap individuals: 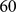 Europeans, 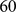 Africans, and 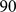 Chinese and Japanese (data from [29]). In these data, the three continental groups are well separated, making interpretation of the results relatively straightforward and selection of an appropriate number of factors simple. (We discuss the issue of selecting an appropriate number of factors later.) We ran SFA and admixture with three factors; since both of these methods involve a numerical optimization we ran each 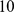 times, using 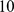 different random starting points, and in each case the results were effectively identical across runs.

**Figure 3:**
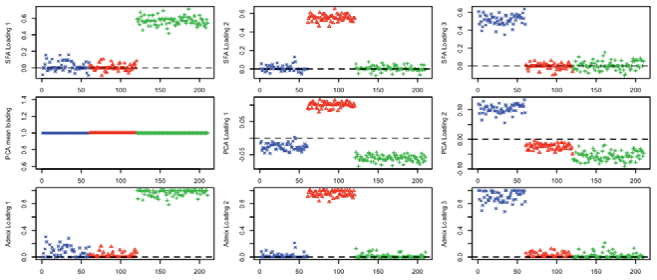
Results of applying SFA, PCA, and admixture to the HapMap genotype data. Each plot shows the estimated loadings (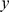-axis) across individuals (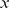-axis). SFA loadings are in the first row, PCA loadings in the second, and admixture loadings in the third. European individuals are denoted with blue ‘x’s, African individuals are denoted with red triangles, and Asian individuals are denoted with green ‘+’s. A dashed horizontal line is at zero on the 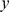-axis.

Figure 3 compares the loadings from SFA and admixture with the first three PCA loadings. All three methods clearly separate out the three groups, but SFA and admixture produce qualitatively different results from PCA. In particular, in SFA and admixture, each individual has appreciable loading on only one of the three factors; from this we infer that the three corresponding factors each represent the allele frequencies of a single continental group. In contrast, in PCA, each individual has appreciable loading on all three factors, and the factors themselves do not have such a straightforward interpretation.

In some ways the different representations obtained by SFA, PCA, and admixture are equivalent: the resulting matrix product, 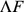, from each method is essentially identical (not shown). However, in this case we view the results of SFA and admixture as more easily interpretable. Specifically, the three SFA and admixture factors correspond to the Asian, African, and European allele frequencies, respectively. In contrast, the first PCA factor corresponds to the overall mean allele frequency, and subsequent factors correspond to other linear combinations of the allele frequencies in each group. These differences are driven by the different constraints on the 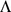 and 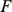 matrices, not by one factorization fitting the data better. Note that, although PCA is forced into using the mean allele frequencies as its first factor by our following the common practice of applying it to the standardized genotype matrix with the genotype means removed, in this case PCA produces almost identical results when applied to the original genotype matrix (results not shown).

One consequence of SFA and admixture factors corresponding to individual group frequencies is that their results are more robust to the number of individuals included from each group. For example, when we removed half of the Africans from the sample and reran the methods, the results from SFA and admixture were essentially unchanged, whereas PCA results changed more appreciably (Figure S1). The intuition here is that, for SFA and admixture, removing some African individuals has only a small effect on the factor corresponding to Africans (because the sample African allele frequencies change slightly) and a negligible effect on the factors corresponding to the European and Asian individuals. These small changes in the factors translate into correspondingly small changes in the loadings for each remaining individual. In contrast, removing half of the Africans changes all three PCA factors: the modified sample has a different overall mean allele frequency (first factor), and this has a cascading effect on subsequent factors and their loadings. Indeed, the general lack of robustness of PCA to sampling scheme is well known [30], [31].

In more complex settings, we have also found SFA and admixture to be more robust than PCA to sampling scheme. We illustrate this using data on 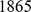 SNPs typed in 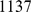 individuals from 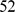 worldwide populations, including the HapMap individuals considered above plus the Human Genome Diversity Panel [29]. These data contain a much higher proportion of individuals with European or Asian ancestry than the HapMap data alone. Analyzing these data with three factors, SFA and admixture produce loadings for the HapMap individuals that are essentially identical to those obtained from the analysis of the HapMap individuals alone (Pearson correlation 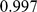 for SFA; 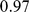 for admixture). In contrast, the corresponding PCA loadings change more substantially (correlation 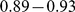).

### Isolation by distance models

We now compare the methods on some simple isolation-by-distance scenarios, involving both one dimensional and two dimensional habitats. For the 1-D habitat we assume 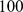 demes equally-spaced on a line, and for the 2-D habitat we assume 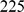 demes arranged uniformly on a 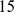 by 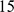 square grid. In each case demes are assumed to exchange migrants in each generation with neighboring demes. We applied PCA, SFA and admixture to data from both 1-D and 2-D simulations.

**Figure 4:**
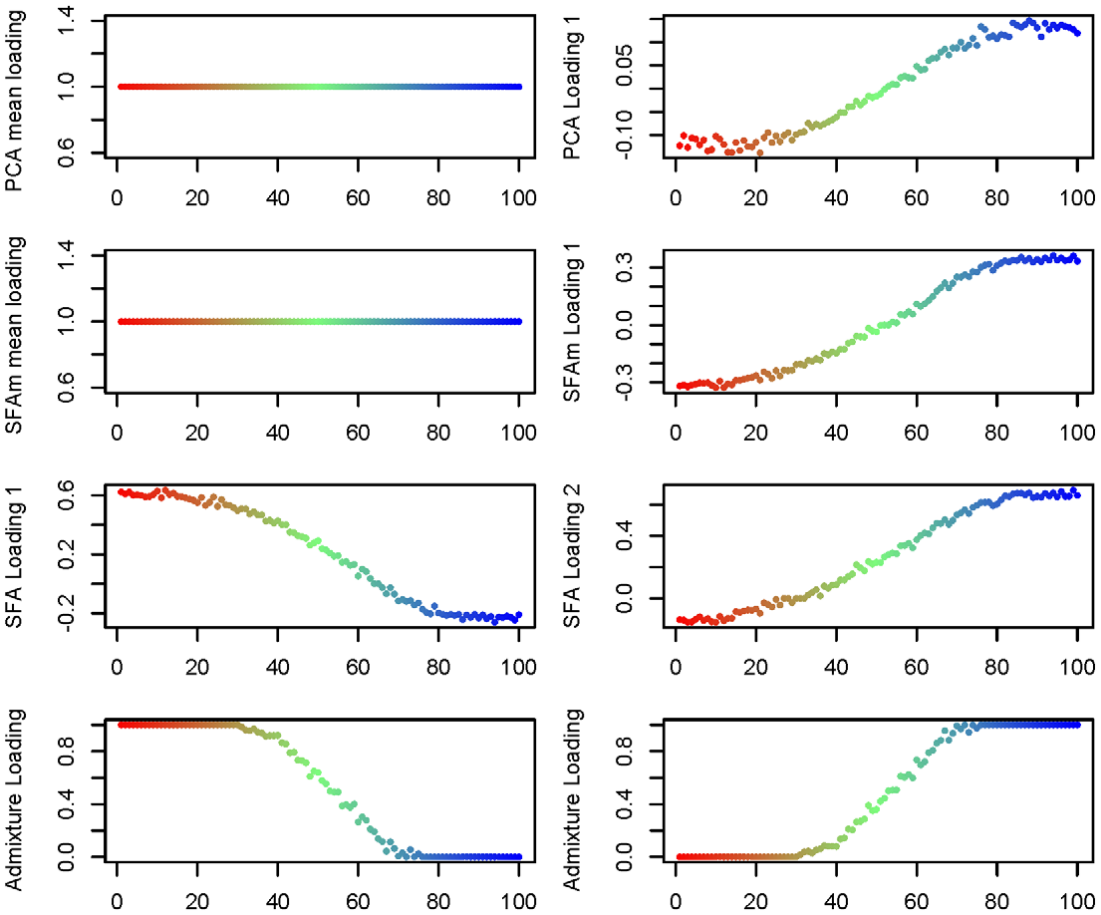
Estimated factor loadings from PCA, SFAm, SFA, and admixture for the 1-D isolation-by-distance simulation. In each plot the individuals are colored and ordered along the 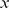-axis by location in the 1-D habitat.

**Figure 5:**
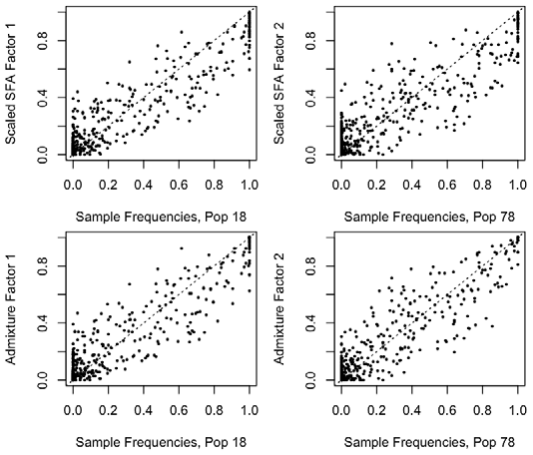
Estimated scaled factors from SFA and admixture on the 1-D isolation-by-distance simulation against the generating allele frequencies. In each plot the factors (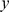-axis) are plotted against the population allele frequencies for the closest-matching population. The SFA factors were truncated to have a minimum of zero and scaled to have a maximum of one. The dashed diagonal line shows 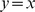.

In the 1-D scenario, for each method, two factors suffice to capture the underlying geographical structure (Figure 4). However, as for the discrete data considered above, the interpretations of the resulting factors differ across methods. In SFA and admixture, the two factors represent, roughly, the allele frequencies near either end of the line (Figure 5). The genotype of each individual along the line is then naturally approximated by a linear combination of these two factors, with weights determined by their position along the line (e.g., individuals near the center of the line have roughly equal weight on the two factors). The loadings in SFA seem to capture the underlying structure slightly better near either end of the line than those from admixture, whose loadings effectively saturate at zero on the first and last third of each line. This may partly reflect the constraint that the admixture loadings must sum to one, but may also be exacerbated by the assumption of a binomial distribution, and in particular the assumption of a binomial variance. In contrast, in PCA, the first factor represents the mean allele frequencies and the second represents a difference between the allele frequencies near either end of the line. Thus PCA represents each individual as the mean allele frequency, plus the allele frequency difference weighted according to the location of the individual relative to the center (the weight being zero for individuals near the center of the line, positive at one end of the line, and negative at the other). Again, this behavior is not solely due to our applying PCA to the standardized genotype matrix: it produces almost identical results when applied to the original genotype matrix (results not shown).

**Figure 6:**
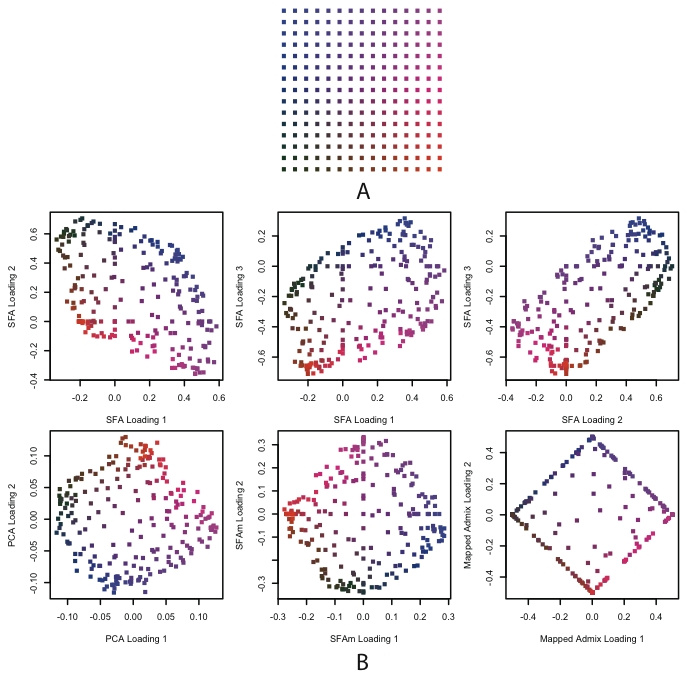
Results of SFA, PCA, SFAm, and admixture applied to simulated genotype data from a single 2-D habitat. In Panel A, each dot represents a population colored according to location. In Panel B, each plot is of the loadings across individuals against each other, where the colors correspond to their locations in Panel A. The first row shows the three SFA loadings against each other from a three factor model. The second row shows the second two PCA loadings, the SFAm loadings, and the mapped admixture loadings (see text for details). All of the methods recapitulate, to a greater or lesser extent, the geographical structure of the habitats (up to rotation).

For the 2-D scenario (Figure 6), the methods differ more substantially in their results. In particular they differ in the number of factors that they need to model the underlying geographical structure.

Due to the convexity constraint, admixture requires four factors, corresponding roughly to the allele frequencies at the four corners of the square habitat. (This result depends on the shape of the habitat; intuitively, the convexity constraint means that admixture needs a factor for each extreme point of a convex habitat.) Even then, the 2-D structure is only easy to visualize after the four factor loadings have been mapped into two dimensions (see Methods). As in the 1-D setting, the loadings for individuals near the edges of the grid saturate near zero or one.

In contrast, both PCA and SFA can capture the structure using three factors, although again they accomplish this in different ways. PCA uses the mean allele frequencies as the first factor, and then two factors that represent deviations from this mean in two orthogonal directions (e.g., the diagonals of the square). As a result the PCA loadings on the second and third factors effectively recapitulate the geography of the space, as previously observed [14], [15], [30].

The results from SFA are more complicated to describe. All three factors represent linear combinations of the allele frequencies on the grid, where the weights of these allele frequencies vary in a consistent way along a particular direction. For example, in the first row of Figure 6B, the first factor has increasing weight as one moves from the bottom to the top of the grid. The result is that the loadings from any two factors recapitulate a skewed version of the geography.

In both of these settings, particularly the 2-D case, the PCA loadings seem to have the simplest interpretation. This is because, after subtracting the genotype mean, the 1-D structure can be captured by a single factor, and the 2-D structure captured by two factors, in each case yielding an attractive geographical interpretation. Thus PCA's use of the mean allele frequency as its first factor, which hinders interpretability in the discrete case, actually aids interpretability in settings with more continuous structure.

However, the use of the mean allele frequencies as the first factor need not be limited to PCA. In particular it is straightforward to modify SFA to behave in a similar way, either by applying it to the genotype matrix with the genotype means subtracted, or by modifying the model to include a mean term (i.e., a factor for which all individuals have loading one). We take the later path here because we think there are advantages to estimating the mean along with the factors, rather than as a preprocessing step. We refer to this approach as SFAm; see Methods for details. Applying SFAm to both the 1-D and 2-D scenarios produces results that are effectively identical to PCA, recapitulating the geographic structure in one or two additional factors respectively (Figure 4 and Figure 6).

In summary, the fact that the first factor in PCA represents the mean allele frequencies is responsible both for the fact that it produces less interpretable factors in the discrete case and more interpretable results in the continuous case. Because SFA provides the flexibility of choice whether or not to include the mean, it can produce interpretable results in both scenarios. Indeed, in the discrete case SFA effectively recapitulates the results of admixture, and in the continuous settings SFAm effectively recapitulates the results of PCA.

#### Mixture of continuous and discrete populations

**Figure 7:**
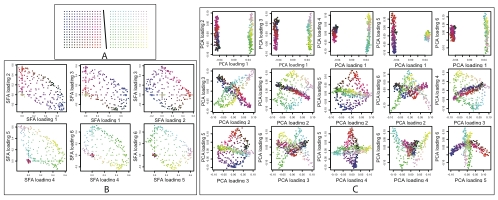
Results on simulated genotype data from a two independent 2-D habitats. In Panel A, each dot represents a population colored according to habitat and location. Colors in Panels B and C indicate locations in Panel A. Panel B shows how SFA captures the structure with a six factor model. Loadings on the first three factors (first row of Panel B) correspond to location in the first habitat; individuals in the second habitat have essentially zero loading on these factors. Similarly, loadings on the other three factors (second row of Panel B) correspond to location in the second habitat. Panel C shows estimated loadings from PCA for the same data. Each plot shows one loading plotted against another. Although the PCA results clearly reflect the underlying structure one might struggle to infer the structure from visual inspection of these plots if the colors were unknown.

To illustrate the potential for SFA to produce new insights in population structure analyses, we now present a hypothetical example for which SFA seems better suited than either admixture or PCA. For this simulation we generated samples from two independent 2-D habitats, so the data have both discrete structure (between the habitats) and continuous structure (within each habitat) (Figure 7A).

We applied PCA, SFA and admixture to these data. Because SFA effectively requires three factors to capture a 2-D structure, we expected it to require six factors to capture this mixture of two 2-D structures, and so we applied SFA with six factors. By analogous reasoning we applied admixture with eight factors.

Reassuringly, SFA behaved as one might predict from the results on discrete and continuous simulations above: three factors were used to represent each of the two 2-D habitats. In particular SFA successfully captured the discrete structure in this case, in that individuals from the first habitat have near-zero loadings on the factors corresponding to the second habitat, and vice versa (Figure 7B). These results were consistent across multiple runs from different random starting points.

In contrast, admixture produced less consistent results from multiple runs (results not shown). In about 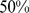 of runs it behaved as we might have hoped, using four factors to represent the corners of each of the two habitats, and effectively capturing both the continuous and the discrete structure. In other cases admixture would converge to alternative solutions, for example using five factors for one habitat and three for another.

PCA produced qualitatively different results, with each individual having a non-zero loading on most factors. The second PCA loading is straightforward to interpret, since it separates individuals from the two habitats. However, subsequent PCA loadings, while jointly capturing the underlying structure, are geometrically beautiful but individually difficult to interpret (Figure 7C).

In this case we view the results from SFA as preferable to those from admixture or PCA. In particular, in a real data analysis, where the underlying structure is unknown, we think that we would more easily deduce the underlying structure (Figure 7A) from the results of SFA (Figure 7B) than from the results of PCA (Figure 7C). However, we could envisage results that are still more interpretable than those from SFA. In particular, one could imagine developing a method (e.g., by appropriate constraints or priors on the matrices) that mimics the results from SFAm or PCA on the single 2-D habitat. That is, one could imagine a method that uses three factors for each 2-D habitat: one factor to be the mean allele frequency, and two factors to capture the geography. Incorporating a single mean term, as do SFAm and PCA, does not achieve this goal because a single mean term does not capture the different mean allele frequencies of the two independent habitats.

### Clustered sampling from a continuous population

Up to now we have avoided discussion of automatic selection of an appropriate number of factors, instead relying on intuition and heuristic arguments to guide this selection. In principle one could attempt to formalize this process within a model-selection framework, since SFA has an underlying probabilistic model. However, automatic selection of an appropriate number of factors is difficult, not least because in many practical applications there does not exist a single “correct” number of factors. For example, our 1-D simulations involved 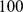 discrete populations exchanging migrants locally, so in some sense a “correct” number of factors is 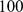, but for realistic-sized data sets reliably identifying 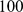 factors will not be possible, and analyzing the data with 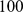 factors is unlikely to yield helpful insights. Note that interpretability of factors does not necessarily correspond with statistical significance: in isolation by distance scenarios many PCA factors may be statistically significant [13], but usually only the first few are easily interpretable, with additional factors representing mathematical artifacts [30]. For these reasons, in practice it can be helpful to run methods such as admixture and SFA multiple times, with different numbers of factors, to see what different insights may emerge. (PCA need only be run once, because adding additional factors does not change existing factors.)

To illustrate these issues we applied the methods to a situation that mimics clustered sampling from a continuous habitat; specifically we used samples of twenty individuals from each of five evenly-spaced demes from the 1-D simulation above. These samples can be represented in either a low-dimensional way, as five clusters along a continuum, or a higher-dimensional way, as five distinct populations.

**Figure 8:**
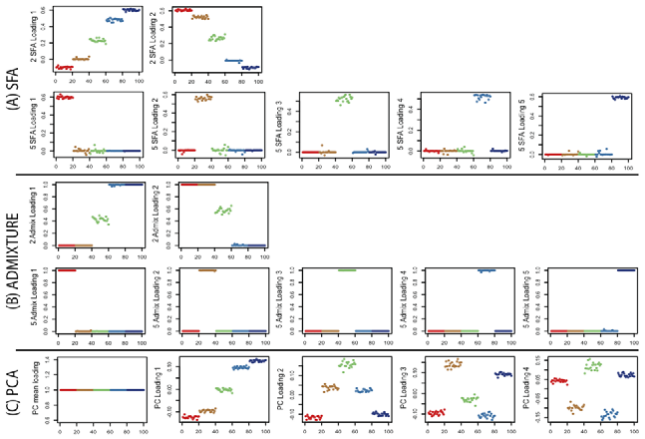
Results from SFA, admixture, and PCA for the clustered 1-D simulation. All plots show the individuals on the 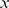-axis (colored and ordered by location with respect to the 1-D clustered isolation-by-distance model) plotted against the estimated loadings.

Applying SFA to these data (Figure 8A), we obtain qualitatively different results depending on the number of factors used: with two factors the SFA loadings represent the five demes as five points along a line (so each factor corresponds, roughly, to the allele frequencies near each end of the line), whereas, with five factors, the SFA loadings separate the five demes into discrete groups (so each factor corresponds to the allele frequencies within a single deme).

Applying admixture to these data (Figure 8B), we obtain similar results as for SFA, except that in the two factor case the five groups are compressed into three groups. Thus, as with the 1-D isolation-by-distance simulations, admixture tends to over-discretize continuous variation.

Applying PCA to these data (Figure 8C), the first two factors capture the continuous variation along the line, as in the 1-D simulations. Subsequent factors each distinguish finer-scale structure among the five demes, and the first five PCA factors, jointly, fully capture the structure. However, each factor is individually difficult to interpret. In particular, because computing additional PCA factors does not affect earlier factors, PCA never reaches a representation in which five factors each represent the allele frequencies of a single deme.

Applying SFAm to these data, with one factor plus the mean term, produces results almost identical to the first two factors of PCA (results not shown).

In summary, this simulation illustrates two important points. First, there is not necessarily a single “correct” number of factors: by applying methods such as SFA and admixture with different numbers of factors, we may obtain qualitatively different results that provide complimentary insights into the underlying structure. Second, SFA seems to be more flexible than either PCA or admixture in its ability to represent both discrete and continuous structure.

### European genotype data

We now compare the three methods on a set of European individuals, consisting of genotype data on 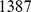 individuals at 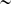
200,000 SNPs (after thinning to remove correlated SNPs). The collections and methods for the Population Reference Sample (POPRES) are described by [32]. Previous analyses of these and similar data using PCA have found that the first two PCA factors recapitulate the geography of Europe (e.g., [14], [15]).

**Figure 9:**
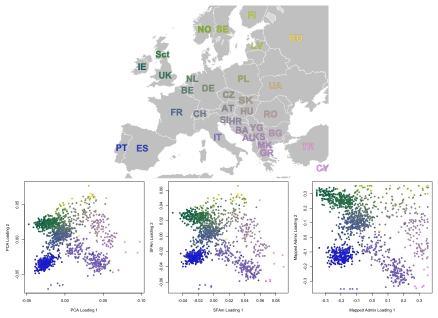
Results from PCA, SFAm, and admixture for the POPRES European data. These results were rotated (but not rescaled) to make the correspondence to the map of Europe more immediately obvious. The results from SFAm are very similar to the results from PCA for these data, effectively recapitulating the geography of Europe.

Based on the results from the 2-D simulations, we chose to apply SFAm (with two factors plus a mean) here, rather than SFA. The results from SFAm are strikingly similar to those from PCA (Figure 9). In a few cases the sparsity-inducing prior we used in SFAm is evident, in that there is a slight tendency for factor loadings near zero to be shrunk closer to zero (appearing as faint diagonal lines of individuals in the rotated SFAm plot). However in general the effect of the sparsity-inducing prior is minimal in these kinds of situations, where the data do not actually exhibit sparsity. Different runs of SFAm produce alternative rotations of this same basic image.

As in the 2-D simulations, admixture with four factors is able to capture the geography, but only after these four factors have been mapped to a two-dimensional space (see Methods). As in the 1-D and 2-D simulations, admixture tends to push the data towards the extremes relative to PCA or SFAm, although this effect is substantially less prominent than in the simulations (perhaps due, in part, to the larger number of SNPs). The ability of admixture-based models to capture geography has been noted before [33].

All three methods are computationally tractable for data sets of this size. Of the three methods, PCA was fastest and admixture was slowest, but all three methods took less than a few hours on a modern desktop.

### Admixture and Indian genotype data

Recall that, in settings with discrete structure, the SFA factors, like the admixture factors, correspond to the allele frequencies of each discrete populations. One consequence of this is that in settings involving admixed groups, the SFA loadings are highly correlated with the admixture proportions of each individual. Indeed, in some settings it is possible to translate the SFA loadings into estimates of admixture proportions. Specifically, if an individual 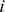 has all positive loadings, and the loading on factor 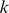 is 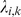, then 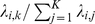 is a natural estimate of that individual's admixture proportion from the population represented by factor 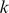. However, this estimate assumes implicitly that factors have all been scaled appropriately, which will only be true if the variance of the allele frequencies in the ancestral populations is similar (something that may well hold in many contexts, but would be difficult to check).

To compare all three methods on real data that appear to involve admixture, we consider the data from a recent study on individuals from India [2]. These data were sampled from 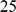 “groups” geographically distributed across India; [2] hypothesized the different groups to be admixed between two ancestral population: ancestral north Indians (ANI) and ancestral south Indians (ASI). This is a challenging data set for admixture analysis because the sample contains no individuals representative of either of the two ancestral populations. For this reason, [2] uses a novel tree-based method (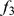 ancestry estimation, described in their supplemental information) to estimate the ancestry proportions of each group.

**Figure 10:**
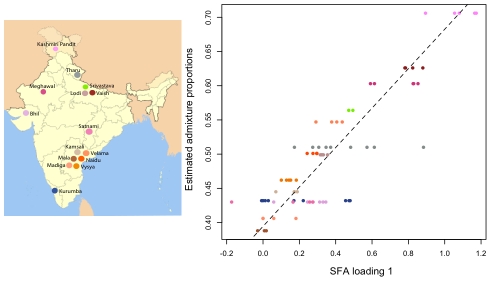
Plot of estimated admixture proportions of each Indian group versus the relative admixture proportions from SFA on the Indian data set. This plot shows good correlation between the relative admixture proportions from SFA and the estimated admixture proportions from previous work. The colors coding the groups are described in the India map.

We applied PCA, SFA with two factors, and admixture with two factors to the genotype data from this study, after imputing the missing genotypes, removing some of the outlier populations as defined in the original study, and removing SNPs with a minor allele frequency less than 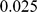 (see Methods). We encountered problems applying SFA to these data with the low frequency SNPs included; specifically, SFA often converged to a solution where one individual had a very small residual variance term. All three methods produce very similar loadings (Figure S2) that correlate well with the ancestry proportions estimated in [2] (Pearson correlations of 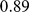 for PCA, 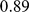 for SFA, and 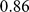 for admixture) (Figure 10).

In one sense, the factor loadings provide more detailed ancestry information than the 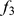 method, because the loadings are individual-specific rather than group-level. However, in this setting, the loadings provide measures of individual-specific ancestry that are reliable only in a relative sense. That is, they may correctly order the individuals in terms of their degree of ancestry in each ancestral population, but do not necessarily provide accurate ancestry proportions for each individual. For example, the estimated ancestry proportions from admixture range from 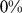 to 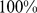, whereas the group-level estimates from the 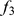 method range from 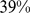 to 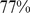. This reflects the difficulty of reliably estimating the ancestral population allele frequencies in the absence of any reference individuals from the ancestral populations.

## Discussion

In this paper we have presented a unified view of the two most common methods to analyzing population structure – admixture-based models and PCA – by interpreting both as matrix factorization methods with different constraints on the matrices. This unification provides insights into the different behavior of these methods under various scenarios. For example, viewing admixture-based models as imposing a convexity constraint explains why these models would be expected to need four factors to capture the structure across a square habitat, whereas PCA requires only two factors plus a mean.

Viewing these methods as special cases of a much larger class of matrix factorization methods also immediately suggests many possible novel approaches to the analysis of population structure. Here we consider one such method, sparse factor analysis (SFA). We illustrate that SFA bridges the gap between PCA and admixture-based models by effectively recapitulating the results from admixture-based models in discrete population settings, and recapitulating the results from PCA in continuous settings. We also illustrate a scenario involving a mixture of discrete and continuous structure where SFA produces more interpretable results than either admixture-based models or PCA.

We have also experimented with two other matrix factorization approaches in the analysis of population structure: sparse principal components (SPC) [24] and non-negative matrix factorization [23]. SPC, implemented in the R function SPC in the R package PMA, computes sparse PCs by solving a penalized matrix factorization problem with an 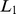 penalty (a penalty on the sum of the absolute values of the factor loadings) to encourage sparsity. The algorithm is greedy in that it computes the factors one at a time, each time removing the effect of the previous factors from the original matrix. The user can choose whether to require the factors to be orthogonal; in our experiments we did not require orthogonality. SPC has a user-defined tuning parameter that controls the level of sparsity. We found that, with careful choice of this parameter, we were able to get SPC to produce results similar to PCA when the data are continuous, and closer to an admixture-based model when the data are from discrete groups. In particular, the main difference from SFA was on the data from two independent 2-D habitats. where SPC did not model the two habitats in separate factors. (We were unable to apply SPC to the larger European and Indian data sets, due to limitations of 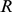.)

As its name suggests, non-negative matrix factorization (NMF) [23], [34] constrains the factors and loadings to have non-negative values. For data sets considered here, we found that NMF typically produced results similar to SFA. However, NMF is less flexible than SFA in that it effectively requires the input matrix to be non-negative. In the genetic context this is not a big limitation as genotype data are most often encoded as non-negative integers (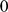, 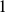, 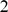), but even here it makes NMF slightly less flexible. For example, this means that NMF cannot be applied to genotype data that have been mean-centered, and there is no sensible way to include a mean term as in SFAm. As we have seen, in some settings incorporating a mean improves the interpretability of the results.

The computational methods used to perform the matrix factorization for PCA, SFA, and admixture (and also *structure*) are practically quite different. In particular, the PCA factorization has a single global optimum that can be obtained analytically, and so multiple runs of PCA produce the same results. In contrast both admixture-based models and the SFA factorizations can have multiple local optima, and the computational algorithms used can produce different results depending on their starting point. In practice, in simple cases (e.g., involving a moderate number of discrete populations), both algorithms appear to produce consistent results across runs. In more complex situations we have found more variability in the results, particularly when the number of factors is large. In some cases there appear to be identifiability issues: for example, in the European data, multiple runs of SFAm produce loadings that are rotations of one another.

Another qualitative difference between the three methods is that PCA produces consistent results as more factors are added, whereas admixture-based methods and SFA may produce qualitatively different results with different numbers of factors. Although consistency may seem a desirable property, there can be benefits to the different perspectives obtained by using different numbers of factors, as we illustrated in the results. To further contrast these two behaviors, consider the application of these methods to data from a continuous 1-D habitat. As noted previously [30], the first PCA loading (after removing the mean) roughly captures position within the habitat, whereas subsequent loadings are sinusoidal functions of increasing frequency. In contrast, when SFA or admixture are run with an increasing number of factors, they redistribute their factors along the line so that each factor represents the average allele frequencies of an increasingly local region. (If too many factors are used, there is not enough signal in the data to differentiate populations on small neighboring segments, and the results become unreliable.) Although the additional factors in each case are qualitatively very different, they simply reflect different ways to capture finer-scale structure in the data. Which of these behaviors is preferable may be context-dependent, but understanding these differences is certainly helpful in interpreting the results of a data analysis.

Although we have focused on the different constraints imposed by different matrix factorization methods, they also differ in another way: their assumed error distribution. In particular, admixture-based models assume a binomial error, whereas PCA is based on a least-squares criterion, which can be interpreted as a Gaussian error, and our SFA explicitly assumes Gaussian error. The binomial error may be more appropriate for data from an admixed population, but in general it is less flexible than the Gaussian model because the binomial variance is determined by the mean, rather than being a free parameter. It seems possible that this partly explains the convergence problems we observed in admixture for the 2-D habitat, in which case it may be worth adapting the admixture model to assume a Gaussian error.

We note that there are several existing approaches to sparse factor analysis besides the novel approach that we introduce here [19]–[21], [35]. Although these methods have similar motivations, they differ in several respects, and we have found that these differences can substantially impact results (not shown). One advantage of our approach is its computational speed. Another feature of our approach is its lack of manually-tunable parameters (other than the number of factors). This, of course, is a double-edged sword, since on the one hand, it makes the method easy to apply, but on the other hand, reduces flexibility. In practice, as our results show, our approach is sufficiently flexible to deal with a range of contexts involving different levels of sparsity.

Our approach to SFA may also be useful in other contexts (e.g., gene expression data [22], [35] or collaborative filtering [36]). In some cases, particularly when the data do not exhibit much sparsity, it may be desirable to extend our method in various ways. For example, as we have implemented it here, SFA encourages sparsity only on the loadings, and in some contexts it may be desirable to encourage sparsity on both the factors and the loadings (as in the general penalized matrix decomposition method [24]). This could be achieved by putting an ARD prior on the elements of 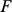, and applying an analog of our ECME algorithm. It may also be fruitful to consider ways to increase the sparsity in the loadings, since in some other contexts we have found that the ARD prior we use can be generous in its use of non-zero loadings. Finally, although we have argued that in the context of population structure that applying methods with different numbers of factors may yield more insight than selecting a single “correct” number of factors, this may not be equally true in all contexts. In particular, the population structure case is complicated by the fact that the factors are often highly correlated with one another (e.g., because they often represent allele frequencies in closely-related populations); in settings where factors are less correlated it may be more helpful to consider methods for automatically selecting the number factors (e.g., [37]).

## Methods

### Genotype simulations

We simulated genotypes from 1-D and 2-D habitats using the program ms [38], using stepping-stone models similar to [30]. In the 1-D model we assumed 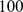 demes along a line and allowing a high level of migration (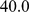) between adjacent demes. This migration rate produced an 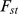 of 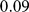 between the two demes at either end of the line, which enables the two most extreme demes to be easily separable with 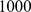 SNPs. We sampled one diploid individual (two independent haplotypes) from each deme at 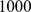 independent SNPs.

For the 2-D simulations, we assumed 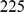 demes arranged in a 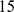 by 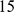 square grid, with migration parameters 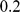 between neighboring demes. We then sampled one diploid individual from each deme at 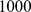 independent SNPs. For the two 2-D habitat simulations, we simulated two independent sets of 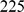 demes and sampled a single individual from each deme at 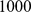 independent SNPs.

For both the simulated and the real genotype data, we encoded each genotype (AA, AB, or BB) as 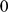, 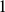 or 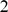.

### POPRES European data

We used the POPRES European data set from [32], and processed the data as in [14]. The POPRES data set was obtained from dbGaP at http://www.ncbi.nlm.nih.gov/projects/gap/cgi- bin/study.cgi?study_id=phs000145.v1.p1 through dbGaP accession number phs000145.v1.p1. This data included 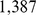 individuals, each of whom identify all four grandparents as being from a particular European country, genotyped at 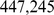 SNPs, and pruned down to 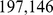 SNPs after removing one of any pair of SNPs that had an 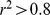 [14].

Since our SFA method does not currently deal with missing data, we imputed missing genotypes using impute2 [39]. We imputed each chromosome by intervals of 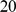
Mb, starting at position 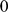, with a buffer of size 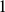
Mb on either side of the interval. We set the number of burn-in iterations to 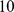 and the number of MCMC iterations to 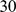. We set the effective population size of the European sample to be 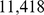, and we used the combined linkage maps from build 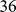, release 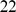 (downloaded from the impute website). We used these imputed genotypes as input to all three methods to facilitate fair comparisons.

### Indian data

We used the Indian genotype data from [2]. The original data includes 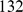 individuals from 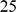 groups; we removed the groups that appeared to be genetic outliers as described in the original paper (Sahariya, Nysha, Aonaga, Siddi, Great Andamanese, Hallaki, Santhal, Kharia, Onge, and Chenchu), leaving 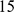 groups and 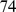 individuals with 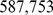 genotyped SNPs. We imputed missing genotypes using impute2 as above, but with an effective population size of 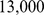, and used these imputed genotypes as input to all three methods. After imputation, we pruned the data down to 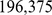 SNPs by removing one of any pair of SNPs that had an 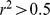, and removing SNPs that had a minor allele frequency less than 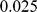.

### Sparse factor analysis

Let 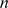 be the number of individuals in a sample and 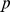 be the number of genotypes. Represent each allele at a locus as a number (e.g., for SNPs from a diploid organism, as in our results above, represent 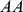 as 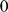, 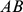 as 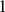, and 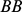 as 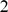). Our factor analysis model with 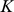 factors can be written as:
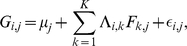
(3)or, equivalently,
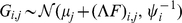
(4)where 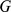 is an 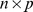 data matrix, 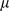 is a 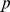-vector of column-specific means, 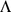 is the 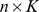 matrix of *factor loadings*, 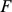 is the 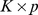 matrix of *factors*, and 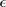 is an 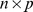 matrix with each element independently distributed 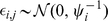. We put a gamma prior on the inverse residual variance that acts as a regularizer: 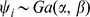, which has mean 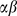 and variance 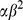. In practice, we set 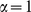 and 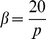. This model, with a mean term, is referred to as *SFAm* in the main text; the SFA model is obtained by fixing the vector 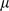 at zero. The ECME algorithm for fitting SFAm is described below; the ECME algorithm for fitting SFA is obtained by simply setting 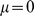 throughout. Note that here we have chosen to have column-specific (i.e., SNP-specific) means and row-specific (i.e., individual-specific) variances 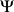. It is possible to modify the ECME updates below to allow for different assumptions, for example to allow row-specific means or column-specific variances. In some contexts, including the population structure problem considered here, it might make sense to allow more general assumptions, such as variance terms on both the rows and columns of the matrix; indeed these options are implemented in the SFA software, although not investigated here.

To induce sparsity in the factor loadings 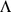, we use an automatic relevance determination (ARD) prior [40]. Specifically, we assume 
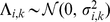
, where the matrix 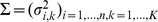 is a parameter that we estimate, together with the other parameters, using maximum likelihood. If the estimate of 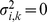, this implies that 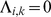, thus inducing sparsity.

Integrating out 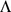, the rows of 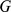 are conditionally independent given the other parameters, with:
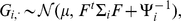
(5)where 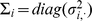 (a diagonal matrix with the 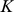-vector 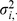 on the diagonal), and 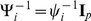. Thus the log marginal likelihood for the parameters 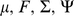 is:
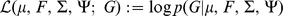
(6)

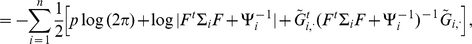
(7)where 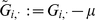.

### Sparse factor analysis ECME algorithm

We fit this model using an expectation conditional maximization either (ECME) algorithm [41] to maximize 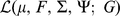. This algorithm is similar to an EM algorithm, but each maximization step maximizes either the expected log likelihood, or the marginal log likelihood, for a subset of the parameters conditional on the others. Specifically, the updates to 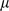, 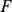, and 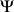 involve maximizing the expected log likelihood (with the expectation taken over 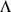), whereas the updates to 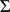 directly maximize the log marginal likelihood.

To compute the expected log likelihood requires the first and second moments of the factor loadings 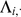. The data 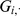 and the loadings 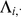 are jointly normal (as in, e.g., [42]):
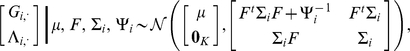
(8)where 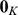 is a 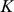-vector of zeros. Standard results for joint Gaussian distributions give the conditional expectation for 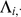:
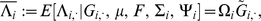
(9)where 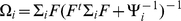. Similarly, the conditional second moment is given by:
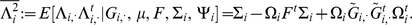
(10)


The updates for 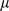, 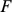, and 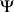 involve maximizing the expected complete data log likelihood, 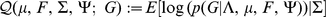, which from Equation 4, and including the prior distribution on 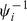, is given by:
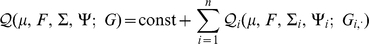
(11)where
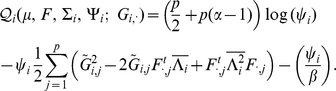
(12)


Taking the derivative of 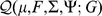 with respect to 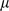 and setting to 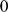, we get the update for 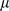:
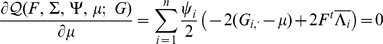
(13)

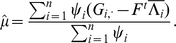
(14)In these expressions, and in what follows, we are assuming element-wise multiplication when a scalar multiplies a vector or a matrix.

Taking the derivative of 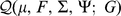 with respect to 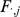 and setting to zero, we get the update for 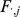:
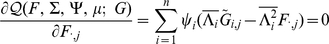


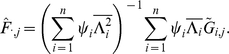
(15)


Taking the derivative of 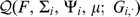 with respect to 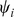 and setting to zero, we get the update for 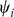:
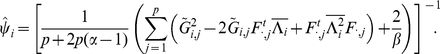
(16)


To update 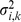 we can use the result from [40] to obtain the values of 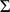 that maximize the log marginal likelihood 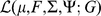 with fixed values of 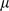, 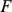, and 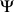:
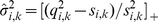
(17)where 
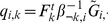
 and 
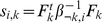
, where 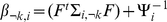 and 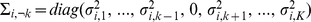. Note that 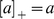 when 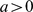 and 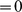 otherwise. This works because, given 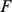, the SFA model (Equation 3) is essentially the sparse regression model considered in [40] with 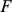 playing the role of the covariates.

Note that 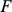 and 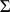 are non-identifiable in that multiplying the 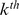 row of 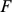 by a constant 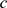 and dividing the 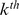 column of 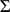 by 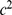 will not change the likelihood (Equation 6). To deal with this we impose an identifiability constraint, 
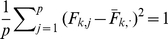
 for 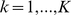, where 
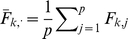
. Specifically, after each iteration we divide every element of 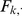 by its standard deviation 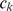, and multiply the 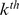 column of 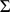 by 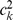.

Because we choose not to update the expected values of the loading matrix 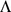 between the CM steps, monotone convergence of the log marginal likelihood is not guaranteed, although in practice it appears to converge well. We find that convergence is reached for the applications described here after fewer than 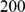 iterations. For each genotype data set, we run SFA multiple times with random seeds, setting the number of factors as described in the text; results presented in figures are a representative example. A C++ package containing the SFA and SFAm code is available for download at http://stephenslab.uchicago.edu/software.html.

### Principal components analysis

For smaller data sets (all but the European and Indian data), we computed principal components by first standardizing the columns of the matrix 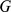 (subtracting their mean and dividing by their standard deviation) and then finding the eigenvectors of the 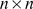 covariance matrix of the individuals in R [43] using the function eigen. In our terminology, these eigenvectors, or principal components (PCs), are the loadings, i.e., the columns of 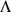. For larger data sets, we identify the PCs using the SmartPCA software from the EigenSoft v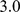 package [7], [13]. For both the European genotype data and the Indian genotype data, we set the number of output vectors to 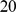, we use the default normalization style, we do not identify outliers, we have no missing data, and we remove all 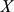 chromosome data.

### Admixture

We ran admixture v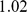 [11] with multiple random starting points using the -s option.

We mapped the four-dimensional admixture proportions into two-dimensions for visualization as follows: the four-dimensional vector 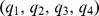 maps to the two-dimensional vector 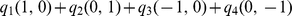.

## Supporting Information

Figure S1 Results of applying SFA, PCA, and ADMIXTURE to the HapMap genotype data after removing half of the Africans. Each plot in the first three columns shows the loadings estimated from the modified data set across individuals. Each plot in the second three columns shows the estimated factors for the original data set against the estimated factors for the modified data set. The first row is SFA, the second row is PCA, and the third row is ADMIXTURE. European individuals are denoted with blue ‘x’s, African individuals are denoted with red triangles, and Asian individuals are denoted with green ‘+’s. A dashed horizontal line is at zero on the y-axis. Note how the correlation of the two unaffected populations for SFA and ADMIXTURE is much higher than for any of the factors in PCA.
(5.76 MB TIF)

Figure S2 Results from PCA, SFA, and ADMIXTURE for the Indian data. Only one estimated loading from SFA and ADMIXTURE are shown because the second set of loadings are perfectly negatively correlated to the first. The results from SFA are almost identical to those from PCA for these data. The individuals are colored as in the map from Figure 10 in the main text according to their population group.
(2.06 MB TIF)

Text S1 Supplemental information. In particular, this information addresses the mathematical consequences of standardizing the genotype matrix before applying a matrix factorization method.
(0.04 MB PDF)
