# Supplementary figures and images for: Analysis of Population Structure: A Unifying Framework and Novel Methods Based on Sparse Factor Analysis
Source: PLoS Genet. 2010 Sep 16;6(9):e1001117. doi: 10.1371/journal.pgen.1001117 (PMC2940725; doi:10.1371/journal.pgen.1001117)

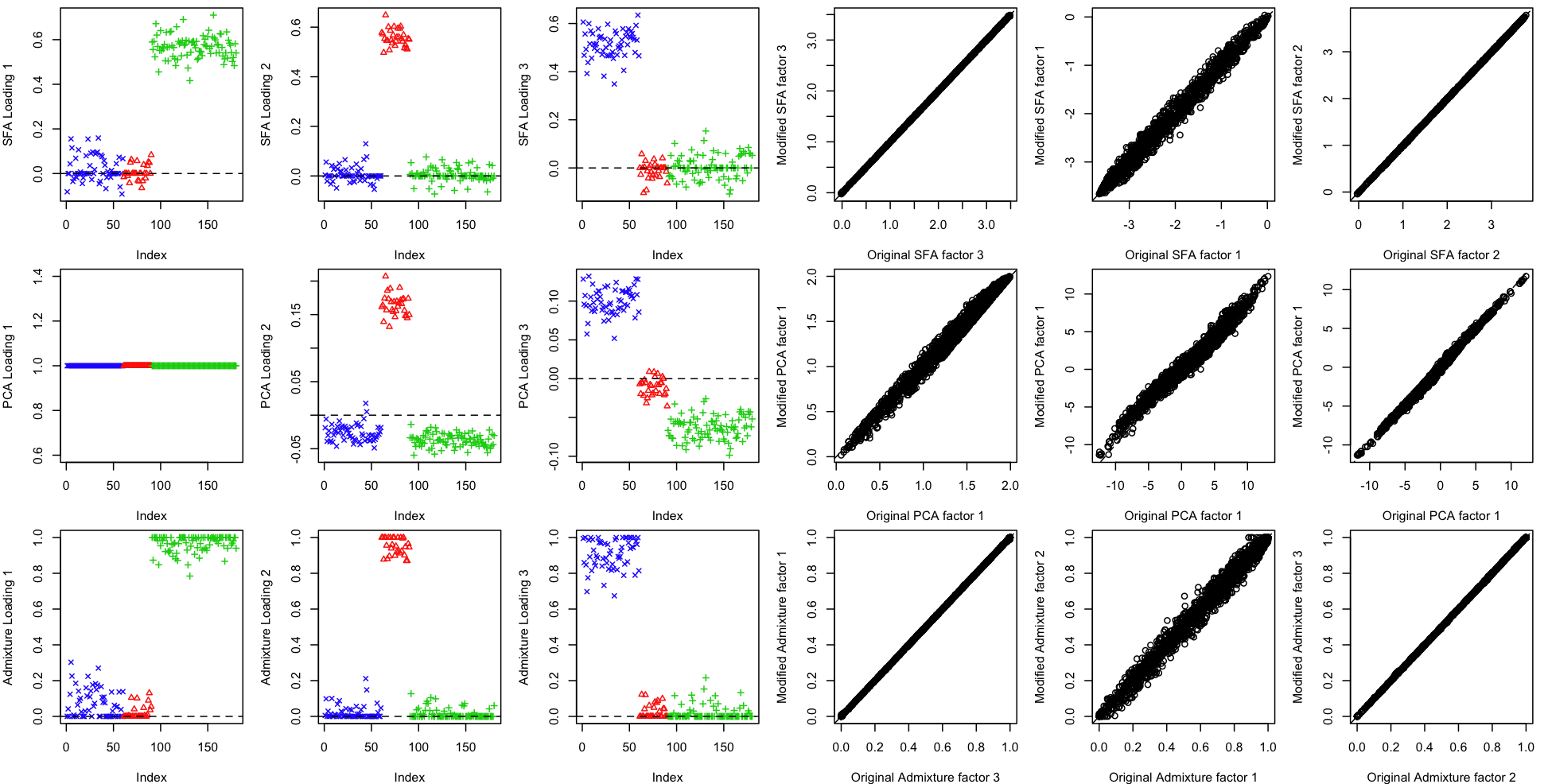

Supplement: Figure S1 — Results of applying SFA, PCA, and ADMIXTURE to the HapMap genotype data after removing half of the Africans. Each plot in the first three columns shows the loadings estimated from the modified data set across individuals. Each plot in the second three columns shows the estimated factors for the original data set against the estimated factors for the modified data set. The first row is SFA, the second row is PCA, and the third row is ADMIXTURE. European individuals are denoted with blue ‘x’s, African individuals are denoted with red triangles, and Asian individuals are denoted with green ‘+’s. A dashed horizontal line is at zero on the y-axis. Note how the correlation of the two unaffected populations for SFA and ADMIXTURE is much higher than for any of the factors in PCA. (5.76 MB TIF) [file pgen.1001117.s001.tif]

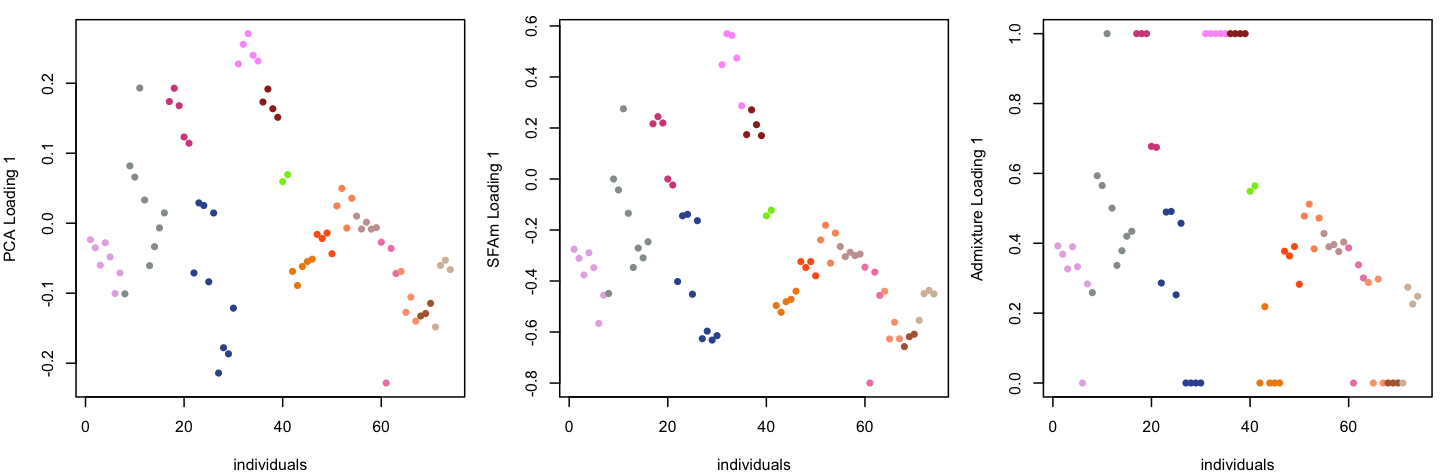

Supplement: Figure S2 — Results from PCA, SFA, and ADMIXTURE for the Indian data. Only one estimated loading from SFA and ADMIXTURE are shown because the second set of loadings are perfectly negatively correlated to the first. The results from SFA are almost identical to those from PCA for these data. The individuals are colored as in the map from Figure 10 in the main text according to their population group. (2.06 MB TIF) [file pgen.1001117.s002.tif]
