# Supplementary material for: Analysis of Population Structure: A Unifying Framework and Novel Methods Based on Sparse Factor Analysis
Source: PLoS Genet. 2010 Sep 16;6(9):e1001117. doi: 10.1371/journal.pgen.1001117 (PMC2940725; doi:10.1371/journal.pgen.1001117)
Supplement: Text S1 — Supplemental information. In particular, this information addresses the mathematical consequences of standardizing the genotype matrix before applying a matrix factorization method. (0.04 MB PDF) [file pgen.1001117.s003.pdf]

SUPPLEMENTAL INFORMATION FOR SPARSE FACTOR ANALYSIS AND POPULATION STRUCTURE

BARBARA E ENGELHARDT, MATTHEW STEPHENS

1. Matrix factorization after standardizing columns of G

Let's consider the difference in matrix G from standardizing each of the columns. Let τ^{-1} be the p-vector of the column standard deviations, and $diag(\tau)$ is the $p \times p$ diagonal matrix of the inverse of those standard deviations.

$$G' = (G - \mathbf{1}_n \bar{G}^t) diag(\tau) \tag{1}$$

where $\bar{G}$ is the p-vector of the column means of G, and G' is the column-standardized matrix to which we apply PCA. Then,

$$(G - \mathbf{1}_n \bar{G}^t) diag(\tau) = \Lambda F \tag{2}$$

$$G = \Lambda F diag(\tau^{-1}) + \mathbf{1}_n \bar{G}^t \tag{3}$$

$$= (\mathbf{1}_n \Lambda) \begin{pmatrix} \bar{G}^t \\ F diag(\tau^{-1}) \end{pmatrix}. \tag{4}$$

This implies that the means play the role of an additional factor that does not necessarily conform to the orthonormal constraint on F. It also implies that the Λ will be scaled (because the F are required to be orthonormal).
